# The Impact of eHealth Literacy on Health Behaviors for Non-communicable Disease Prevention Among University Students in Japan and Indonesia

**DOI:** 10.7759/cureus.78450

**Published:** 2025-02-03

**Authors:** Yayoi Shoji, Andi Masyitha Irwan, Ryota Ochiai, Syahrul Syahrul, Eriko Shinohara, Andi Muhammad Fiqri, Shoko Takeuchi, Erfina Erfina, Mariko Iida, Ariyanti Saleh, Fusae Moriguchi, Sachiyo Nakamura, Yuka Kanoya

**Affiliations:** 1 Global Cooperation Institute for Sustainable Cities, Yokohama City University, Yokohama, JPN; 2 Faculty of Nursing, Hasanuddin University, Makassar, IDN; 3 Department of Nursing, Graduate School of Medicine, Yokohama City University, Yokohama, JPN; 4 Graduate School of Health Care and Nursing, Juntendo University, Urayasu, JPN; 5 Faculty of Nursing at School of Medicine, Tokai University, Isehara, JPN

**Keywords:** covid-19, cross-sectional study, ehealth literacy, health behaviors, university students

## Abstract

Background

Non-communicable diseases account for 71% of global deaths. Health behaviors are fundamental for prevention. Previous studies have indicated that health literacy, such as functional, interactive, and critical, affects people’s health behaviors. Health information is widely available online. Young people tend to access the Internet for information, which highlights the importance of eHealth literacy. Although previous studies have assessed eHealth literacy in single-country contexts, cross-cultural comparisons between high- and middle-income nations remain limited, particularly in understanding its role during global health crises. The COVID-19 pandemic has dramatically accelerated the shift toward seeking digital health information, fundamentally changing how students access and utilize health resources. However, whether eHealth literacy could mitigate the impact of COVID-19 on lifestyle remains unclear. Therefore, this study aimed to examine the impact of eHealth literacy on health behaviors among university students in Japan and Indonesia providing unique insights into cross-cultural differences.

Methodology

This cross-sectional study used a self-administered, web-based questionnaire. The participants were first- to fourth-year nursing students from universities in both countries. eHealth literacy and health behaviors were evaluated via the e-Health Literacy Scale (eHLS) and Healthy Lifestyle Scale for University Students (HLSUS), respectively. Structural equation modeling was conducted to evaluate the impact of eHLS on HLSUS, adjusting for potential confounders. A multigroup structural equation model was used to assess configural, metric, and scalar invariance.

Results

In total, 99 and 407 responses were obtained from Japan and Indonesia, respectively (response rates: Japan, 23.9%; Indonesia, 89.6%). The direct effect of eHLS on HLSUS was 0.33; the indirect effect via the impact of COVID-19 was 0.06. The metric invariance, although not scalar, was supported in the model that included data from both countries.

Conclusions

eHealth literacy universally could influence health behaviors among Japanese and Indonesian university students. However, it did not fully mitigate the effects of COVID-19. Enhancing eHealth literacy could support better health behaviors among university students.

## Introduction

The World Health Organization (WHO) defines non-communicable diseases (NCDs) as conditions that share common risk factors, such as unhealthy diets, physical inactivity, tobacco use, and excessive alcohol consumption, which can be prevented through lifestyle modifications [1]. NCDs are the leading cause of death worldwide, representing a significant global health concern. WHO reports indicate that NCDs claim 41 million lives annually, accounting for 74% of global deaths [1]. Although NCDs were previously considered to primarily affect high-income countries, they now pose a substantial burden in terms of both mortality and morbidity in low- and middle-income countries [2].

Historically, infectious diseases and perinatal mortality are the primary causes of death in low- and middle-income countries [3]. However, in middle-income Asian countries experiencing rapid economic growth, NCDs have become the leading cause of death. Alongside the burden of infectious diseases, the prevalence of NCDs is increasing, creating a dual health challenge [4]. In South Asian countries, the incidence and mortality rates of infectious diseases are declining, while the incidence of NCDs is significantly rising [5,6].

Age-stratified data indicate that 29% of NCD-related deaths occur in the 30-69-year age group [1]. The International Social Security Association predicts that NCD-related mortality will increasingly affect younger age groups in the future [7]. University years are a critical period for the formation of health risk behaviors, such as alcohol consumption and smoking, and an increase in the incidence of NCDs has been observed among younger populations [8-10]. Studies on NCD-related health behaviors among university students have revealed a lack of proactive attitudes toward prevention, insufficient knowledge, and problematic health behaviors. Many students perceive their lifestyle as being undesirable, and those who consider themselves unhealthy report experiencing negative emotions such as apathy, anger, and anxiety [11,12]. These findings highlight the need for NCD prevention programs targeting university students; furthermore, in healthcare students, improving personal health behaviors is crucial for their future responsibilities as healthcare providers.

Health literacy is defined as the ability to find, understand, and utilize information and services to make health-related decisions for oneself and others [13]. Nutbeam categorizes health literacy into functional skills (including reading comprehension and information understanding), interactive skills (encompassing communication and social interaction), and critical skills (involving critical analysis of information) [14]. A 2011 meta-analysis revealed that low health literacy was associated with poorer health behaviors, including increased hospitalization, emergency care utilization, and inadequate medication adherence [15]. Among college students, health literacy is significantly influenced by socioeconomic factors such as living costs, residential area, household income, and parents’ education and occupation [16]. Additionally, demographic factors, including age, sex, field of study, and smoking status, have been found to play a role, with female students and those in health-related fields generally exhibiting higher health literacy [17].

Recently, eHealth literacy has gained prominence in the field of health literacy. eHealth literacy refers to the ability to seek, understand, evaluate, and apply health-related information from electronic sources [18,19]. Previous studies have demonstrated a positive correlation between health-related behaviors and eHealth literacy [20]. Studies have suggested that eHealth literacy influences students’ health behaviors [21,22]. This field has been developed through stages focusing on evaluation, the correlation with health-promoting behaviors, influencing factors, and intervention methods. Future research is expected to address challenges such as the standardization of evaluation methods and the development of personalized treatment strategies [23]. For many university students who have grown up with ubiquitous Internet access, the Internet is a readily accessible information source. However, the abundance and diversity of information available online raises concerns about reliability and bias.

The COVID-19 pandemic has fundamentally transformed the digital health landscape, accelerating advancements in digital healthcare through big data, artificial intelligence, cloud computing, and 5G technologies [24]. This rapid digital transformation has not only been crucial for pandemic control but has also permanently altered how individuals, particularly young adults, access and utilize health information. The importance of health promotion behaviors and eHealth literacy continues to escalate [25]. eHealth literacy is thought to be associated with health behaviors for NCD prevention among younger age groups. However, cross-cultural studies examining these relationships in different economic contexts remain limited, particularly in the post-pandemic digital health landscape.

Although studies investigating this association have been conducted in high-income Asian countries, such as China and Taiwan, similar studies are lacking in middle-income countries such as Indonesia. To address this research gap and gain insights applicable to diverse economic settings, investigating these factors in both high- and middle-income Asian countries is vital. Understanding the current state of eHealth literacy and its relationship to health behaviors is essential to develop programs aimed at improving health behaviors among younger populations. This study aimed to examine NCD prevention-related health behaviors and their associated factors, focusing on eHealth literacy among nursing students in Japan and Indonesia. By analyzing health behaviors and eHealth literacy-related factors among nursing students in Japan and Indonesia, representing average high- and middle-income countries, respectively, among the 48 sovereign states in Asia [26], this study aimed to provide foundational data for developing health behavior improvement programs for NCD prevention.

## Materials and methods

Research design

This cross-sectional observational study used anonymous self-administered questionnaires. Surveys were conducted between February and March 2022 in Japan and between January and February 2023 in Indonesia.

Participants

The study population comprised 413 students from Japan and 454 students from Indonesia, enrolled in years one to four of nursing courses at one university in each country. The nursing departments were selected because of the similarities in their four-year educational programs, which facilitated comparability between the two universities. The inclusion criteria for this study were students enrolled in the nursing departments of Yokohama City University (Japan) and Hasanuddin University (Indonesia) who provided consent to participate in the study. Those with difficulties in reading or writing in the language of the survey were excluded.

The target sample size was determined based on the COnsensus-based Standards for the selection of health Measurement Instruments (COSMIN) four-point rating system, which recommends an “excellent” sample size of at least seven times the number of items and a minimum of 100 participants [27]. After estimating a response rate of 25%, we aimed to distribute the survey to over 400 students from each institution in Japan and Indonesia. Recognizing potential cultural differences in survey participation, we aimed to oversample Indonesian students to ensure adequate power due to anticipated lower response rates in Japan.

Data collection

A web-based questionnaire was distributed to eligible participants, who were invited to participate in the study. Recruitment was performed via an invitation sent to students at universities in both Japan and Indonesia. The eligible participants were informed about the purpose and confidentiality of the study. The respondents were instructed to complete the survey individually without consulting with others or referring to external sources. No participants were specifically excluded during the recruitment process. The completed questionnaires were collected online.

Survey items

Participant Backgrounds

Based on previous literature [28-31], the following key influencing factors related to NCD health behaviors as basic attributes were included: university affiliation, year of study, age group, sex, living arrangement (living alone or with others), health status, body mass index (BMI), and religious restrictions.

Impact of COVID-19 on the Daily Lifestyle

Health behaviors for NCD prevention were considered the primary outcome measure in this study. Given that this study was conducted during the pandemic, we investigated changes in living conditions and behaviors before and after the COVID-19 outbreak as background factors for better understanding their influence on NCD-related health behaviors. The questionnaire comprised 11 items covering various aspects of living conditions and behaviors, including work participation, extracurricular activities, and health-related behaviors such as exercise, nutrition, and smoking habits. The content was specifically created to capture changes in these aspects before and after the COVID-19 outbreak, providing a comprehensive overview of the impact of the pandemic on daily lifestyle and health behaviors for NCD prevention.

Health Behaviors

Healthy Lifestyle Scale for University Students (HLSUS) and e-Health Literacy Scale (eHLS): We employed the eHLS [32] to evaluate participants’ ability to find, understand, and use online health-related information. In this study, we used the Japanese and Indonesian versions of the scale. This scale measures eHealth literacy across three dimensions, namely, functional, interactive, and critical. It comprises 12 questions rated on a five-point Likert scale (never, rarely, sometimes, usually, always). Scores range between 12 and 60, with higher scores indicating higher eHealth literacy. The scale’s reliability and validity have been established in studies with university students by its developers. Permission was obtained from the original authors to create the Japanese and Indonesian versions. The scale was originally developed in Chinese and has since been validated with Taiwanese university students. The reliability and validity of the Japanese and Indonesian versions have also been confirmed [33].

Furthermore, we utilized the HLSUS to evaluate health behaviors [34]. In this study, we translated and culturally adapted the HLSUS into Japanese and Indonesian to ensure its applicability in both contexts. This scale was designed to evaluate the health behaviors of university students across eight dimensions, namely, exercise behavior, regular life behavior, nutritional behavior, health risk behavior, health responsibility, social support, stress management, and life perspective. Questionnaire items encompassed NCD risk factors, such as exercise, diet, smoking habits, alcohol consumption, sleep, and stress. The scale comprises 38 items rated on a five-point Likert scale (never, rarely, sometimes, usually, always). Scores range between 38 and 190, with higher scores indicating better health behaviors. The scale’s reliability and validity have been established in studies including university students by its developers [34]. Permission was obtained from the original authors to create the Japanese and Indonesian versions.

Regarding this study population, we conducted an item analysis using ceiling and floor effects and corrected item-total (I-T) correlations. Construct validity was confirmed using convergent and discriminant validity through multi-trait analysis. Multi-trait scaling analysis supported the construct validity, with scaling success rates and reliability evaluated separately for the Japanese and Indonesian samples.

In the Japanese sample, scaling success rates ranged between 33% and 100%. Cronbach’s alpha for the overall scale was 0.86, with individual factor coefficients ranging between 0.20 and 0.81. In the Indonesian sample, scaling success rates were 100% for all but one factor. Cronbach’s alpha for the overall scale was 0.85, with individual factor coefficients ranging between 0.13 and 0.77.

Based on these results, the scale was deemed to have a certain level of reliability and validity for measuring eHealth literacy across both Japanese and Indonesian populations, while acknowledging some variation in individual factor reliability.

Statistical analysis

Structural equation modeling (SEM) was used to examine the relationships among eHLS, HLSUS, and the pandemic’s impact. HLSUS was the main dependent variable, whereas year of study, age, sex, living arrangements (living alone or with others), health status, BMI, and religious restrictions were treated as independent variables. We constructed multiple models, adjusting for these variables. The model with the best fit was selected.

Model fit was assessed using the following indices: chi-square (χ²), goodness of fit index (GFI), adjusted goodness of fit index (AGFI), comparative fit index (CFI), Akaike information criterion (AIC), root mean square error of approximation (RMSEA), and standardized root mean residual (SRMR). A smaller χ² value indicated a better fit. GFI, AGFI, and CFI values >0.95 and RMSEA values <0.05 indicated a good model fit. Lower AIC values indicated a better fit. Models with GFI, AGFI, and CFI values >0.90 and RMSEA values <0.10 were considered acceptable [35].

Multi-group simultaneous analysis was performed to evaluate the scale’s robustness and determine whether different populations could be interpreted using the same model, anticipating potential differences in path coefficients between Japanese and Indonesian nursing students. The multi-group analysis involved stepwise model constraints implementing two models: the configural model, which assumes similar model structures across different groups, and the metric model, which assumes both similar model structures and equal path coefficients.

The fit of each model in the multi-group analysis was evaluated using RMSEA, CFI, SRMR, and AIC, applying the same criteria used in the country-specific analyses. Acceptable model fit was indicated by a RMSEA < 0.08, CFI ≥ 0.90, and SRMR < 0.05. The AIC evaluates model fit by balancing model complexity and fit to the data. The model with the lowest AIC value was considered the best fit. SPSS 28.0.1.0 (IBM Corp., Armonk, NY, USA) was used for statistical analyses (with a significance level of p < 0.05 for all tests). Confirmatory factor and multi-group simultaneous analyses were performed using AMOS 29 (IBM Corp., Armonk, NY, USA).

Ethical considerations

This study was approved by the ethics committees of Yokohama City University (approval number: A210100029; approval date: February 2, 2021) and Hasanuddin University (approval number: UH23010022; approval date: January 11, 2023). The research collaboration proposal outlined the web-based survey’s objectives, estimated time commitment, and ethical considerations in detail. The participants provided informed consent digitally through the web-based questionnaire.

## Results

Participant characteristics

Participant characteristics are presented in Table 1.

**Table 1:** Characteristics of the study participants.

	Item	Japan	Indonesia
		n (%)	n (%)
Gender	Female	98 (99.0%)	373 (91.6%)
	Male	1 (1.0%)	34 (8.4%)
Year of study	First year	20 (20.2%)	106 (26.0%)
	Second year	21 (21.2%)	113 (27.8%)
	Third year	38 (38.4%)	99 (24.3%)
	Fourth year	20 (20.2%)	89 (21.9%)
Age	17 years old	0	5 (1.2%)
	18–19 years old	19 (19.2%)	176 (43.2%)
	20–21 years old	57 (57.6%)	199 (48.9%)
	22–23 years old	19 (19.2)	27 (6.6%)
	24 years old and over	4 (4.0%)	0
Religious restrictions	Yes	4 (4.0%)	345 (84.8%)
	No	95 (96.0%)	62 (15.2%)
Health status	With underlying disease(s)	12 (12.1%)	57 (14.0%)
	No underlying disease	87 (87.9%)	350 (86.0%)
Living arrangements	Living with parents	70 (70.7%)	165 (40.5%)
	Not living with parents	29 (29.3%)	242 (59.5%)
Body mass index	Underweight/Normal weight	97 (98.0%)	355 (87.2%)
	Obese 1–4	2 (2.0%)	52 (12.8%)

Japan

Valid responses were received from 99 participants (response rate: 23.9%). The majority of the participants were females (female, 99%; male, 1%), and most were aged 20-21 years (57.6%), followed by those aged 18-19 and 22-23 years (19.2% each). Regarding the year of study, 20 (20.2%), 21 (21.2%), 38 (38.4%), and 20 (20.2%) were first-, second-, third-, and fourth-year students, respectively.

Indonesia

Valid responses were received from 407 participants (response rate: 89.6%). The majority of the participants were females (female, 91.6%; male, 8.4%), and almost half were aged 20-21 years (48.9%), followed by those aged 18-19 (43.2%) and 22-23 (6.6%) years. Regarding the year of study, 106 (26%), 113 (27.8%), 99 (24.3%), and 89 (21.9%) were first-, second-, third-, and fourth-year students, respectively.

Score distribution and reliability/validity of each scale

eHLS Score Distribution

The reliability and validity of the Japanese and Indonesian versions of the eHLS have been previously reported, demonstrating good factor validity, cross-cultural validity, and internal consistency. The eHLS score distribution is presented in Table 2. In this study, Cronbach’s alpha coefficients were 0.904 for Japan, 0.844 for Indonesia, and 0.863 overall, indicating high internal consistency.

**Table 2:** Score of the Japanese and Indonesian versions of the eHLS. eHLS: e-Health Literacy Scale

eHLS (3-factor, 12-item)	Japan		Indonesia	
	n = 99		n = 407	
	Mean	SD	Mean	SD
1. I cannot understand the symbols (such as BMI, Body Mass Index) and wording about health information (Reversal item)	4.09	0.69	4.12	0.79
2. I find online health information difficult to understand (Reversal item)	4.03	0.80	3.97	0.67
3. I find the mathematical formulas provided in online health information difficult to calculate. (e.g., the algorithm of calorie consumption, BMI) (Reversal item)	4.01	0.89	3.70	0.81
4. I can locate health information efficiently through search engines	3.65	0.98	3.94	0.62
5. I pay attention to and obtain new knowledge about online health information	3.46	0.97	4.02	0.58
6. I know how to get what I need from online health information	3.71	0.94	4.11	0.59
7. I understand the online health information I have obtained	3.98	0.74	3.98	0.56
8. I will think about whether the online health information applies to my situation	3.95	0.85	3.89	0.61
9. I try to find different sources to verify the credibility of health information	3.87	1.04	4.07	0.59
10. I evaluate the validity and reliability of online health information	3.98	0.87	3.69	0.64
11. I browse various discussions and make a decision or action that is good for health	3.63	1.04	3.74	0.60
12.When I have questions or doubts about online health information, I use other channels to verify the information	3.73	0.92	4.01	0.59

HLSUS Score Distribution

The reliability and validity analyses of both country versions using this study’s data failed to replicate the original eight-factor structure. Several subscales had low alpha coefficients. Owing to the inability to confirm the reliability of multiple items, only the total score was used. The HLSUS score distribution is presented in Table 3. The Cronbach’s alpha coefficients were 0.859 for Japan, 0.851 for Indonesia, and 0.863 overall, demonstrating high internal consistency. The alpha coefficient for both countries was 0.846.

**Table 3:** Scores by domain for the Japanese and Indonesian versions of the HLSUS. HLSUS: Healthy Lifestyle Scale for University Students; SD: standard deviation

HLSUS (8-factor, 38-item)	Japan		Indonesia	
	n = 99		n = 407	
	Mean	SD	Mean	SD
Exercise_behavior	11.5	3.01	12.95	2.07
Regular_behavior	13.55	3.43	12.3	3.15
Nutrition_behavior	14.64	3.21	13.58	2.68
Healthrisk_behavior	9.75	2.09	14.88	1.91
Health_responsibility	24.41	3.06	24.15	3.03
Social_support	21.86	3.54	24.29	2.91
Stress_management	18.96	3.06	18.18	3.3
Life_appreciation	18.1	3.67	20.03	2.73

Impact of eHLS on HLSUS

**Figure 1:**
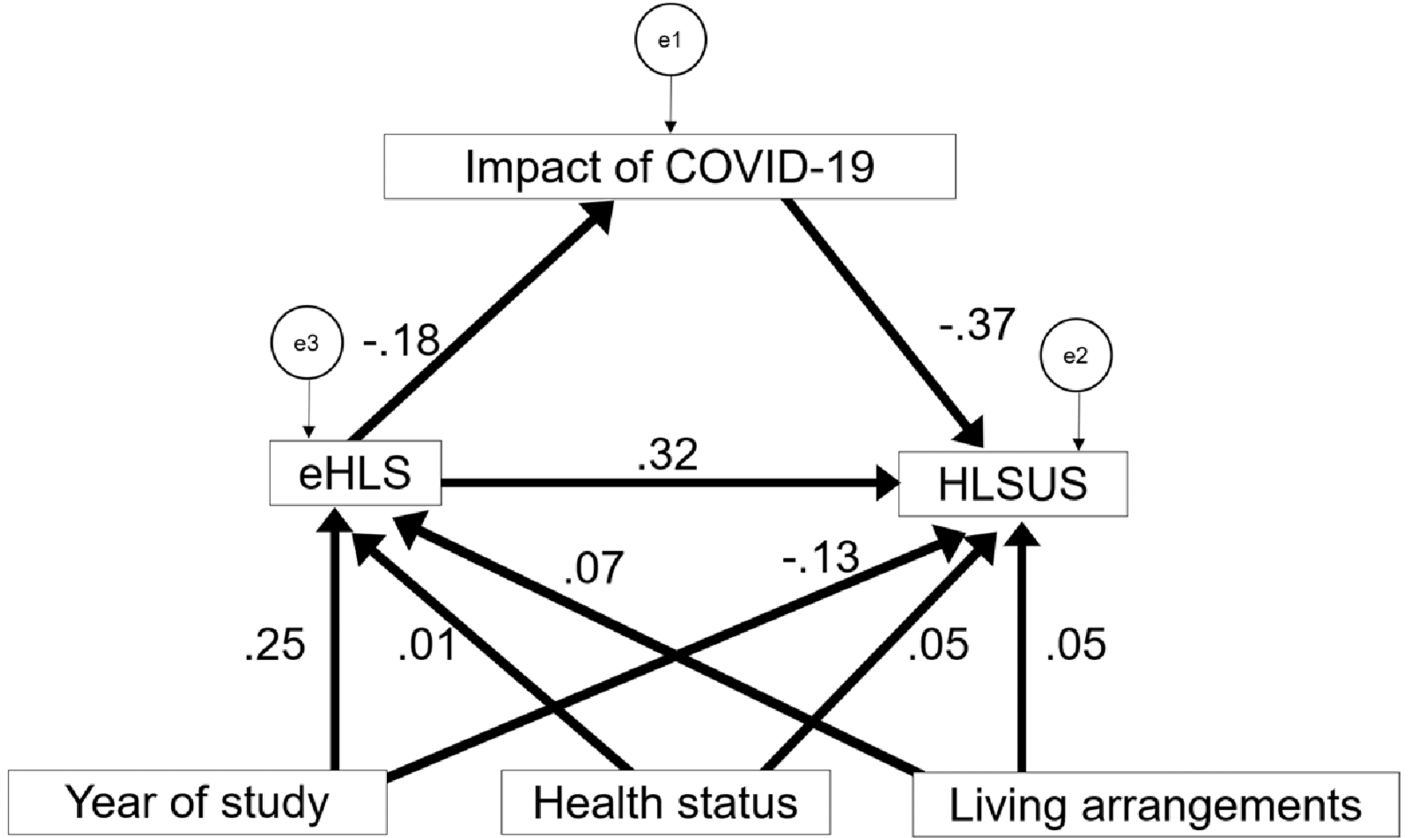
Structural equation model. eHLS: e-Health Literacy Scale; HLSUS: Healthy Lifestyle Scale for University Students

The primary objective of this study was to assess the relationship between eHLS and HLSUS. The results using the SEM analysis showed a significant positive association between eHLS and HLSUS (β = 0.323). Using SEM, the findings revealed that the model with HLSUS as the dependent variable and year of study, health status, and living arrangements as the adjustment variables had the highest fit. Sex and religious restrictions were not included as adjustment variables due to significant response bias. The results are shown in Figure 1. The model fit indices were satisfactory (GFI = 0.996, CFI = 0.988, AGFI = 0.985, RMSEA = 0.011, AIC = 36.340).

The year of study showed a significant negative direct association with HLSUS (β = -0.128, p = 0.001), indicating that for each year increase in academic study, the HLSUS score decreased by 0.128 standard deviation units. A significant positive correlation was found between the academic year and eHLS (β = 0.249, p < 0.001), suggesting that each year increase in the academic study was associated with a 0.249 standard deviation unit increase in the eHLS score. Furthermore, eHLS showed a significant positive association with HLSUS (β = 0.323, p < 0.001), implying that one standard deviation unit increase in eHLS was associated with a 0.323 standard deviation unit increase in the HLSUS score.

Additionally, an indirect relationship was observed where eHLS had a significant negative association with post-pandemic impact (β = -0.183, p < 0.001), which, in turn, had a significant negative association with HLSUS (β = -0.369, p < 0.001). Health status showed no significant positive associations with HLSUS (β = 0.055, p = 0.146) and eHLS (β = 0.010, p = 0.822). Similarly, living arrangements demonstrated no significant positive associations with HLSUS (β = 0.054, p = 0.154) and eHLS (β = 0.067, p = 0.120).

**Figure 2:**
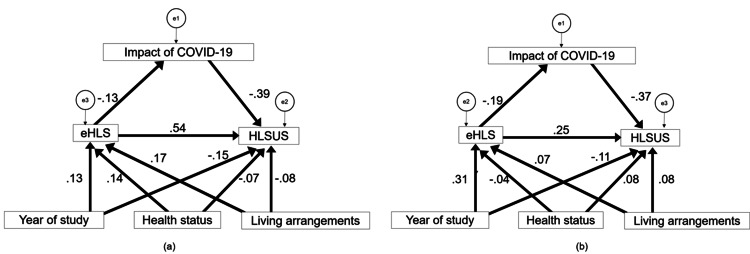
A multi-group structural equation model (configural) comparing Japan (a) and Indonesia (b). eHLS: e-Health Literacy Scale; HLSUS: Healthy Lifestyle Scale for University Students

Subsequently, a multi-group simultaneous analysis was conducted. First, an SEM was performed without constraints by incorporating data from both countries into the same dataset (Figure 2). The model fit indices were RMSEA of 0.054, CFI of 0.884, and SRMR of 0.0299, indicating a relatively good fit despite the CFI not reaching the criterion. This confirms a common model where eHLS influences the HLSUS in both countries. Next, to verify the equivalence of path coefficients, full metric invariance was tested. The model fit evaluation revealed a decrease in CFI to 0.859 and SRMR to 0.0635, failing to confirm full metric invariance. While the configural invariance model was within acceptable limits, the full metric invariance was rejected. The comparison is presented in Table 4. Attempts to establish partial metric invariance did not yield well-fitting models.

**Table 4:** Summary of the multi-group structural equation model. RMSEA: root mean square error of approximation; AIC: Akaike information criterion; CFI: comparative fit index; SRMR: standardized root mean residual

	RMSEA	AIC	CFI	SRMR
Configural	0.054	116.123	0.884	0.0299
Full metric	0.048	112.716	0.859	0.0635

## Discussion

The findings of this study indicated that enhancing eHealth literacy may be associated with the promotion of health behaviors among university students; however, this may not fully mitigate the impact of COVID-19. This study demonstrated that higher eHLS scores were associated with higher HLSUS scores. Although the effect of eHealth literacy on mitigating the impact of COVID-19 was limited, it showed a universal influence on the health behaviors of both Japanese and Indonesian university students. This suggests that improving eHealth literacy might lead to better health behaviors and should be considered when developing health behavior improvement programs for NCD prevention.

Participant characteristics

A notable difference was observed in response rates between Japan (23.9%) and Indonesia (89.6%). The low response rate in Japan may be attributed to the shift to online classes during the pandemic, potentially causing the survey request to be overlooked amid numerous email communications. Additionally, pandemic-related restrictions on accessing university facilities and social changes may have contributed to the low response rate.

Regarding religious prohibitions, only 4% of the Japanese students reported having restrictions compared to 84.4% of the Indonesian students. This difference reflects not only the predominance of Islam in Indonesia, which includes dietary and lifestyle restrictions, but also the broader influence of religion on daily life and social practices. In contrast, in Japan, with a diverse religious landscape, individuals do not have the pervasive impact of religious doctrines on their everyday lifestyle choices. Religious practices in Japan, such as Shinto and Buddhism, tend to emphasize spiritual observance over strict lifestyle constraints, which may explain the relatively low percentage of students reporting religious restrictions. The cultural differences between the two countries, particularly in terms of dietary habits, fasting practices, and social behaviors, further highlight the importance of understanding how religion shapes health behaviors. In Indonesia, Islamic teachings often dictate not only dietary choices (e.g., halal food) but also aspects of physical health, such as fasting during Ramadan, which could influence students’ eating patterns and overall wellness. In contrast, Japan’s secular or less strict religious practices do not similarly dictate everyday health-related decisions to the same extent, offering a wider array of lifestyle choices to young people. Previous studies have highlighted the importance of considering the role of religion in health promotion among young people [36]. The disparity in responses due to religious restrictions suggests that tailored interventions, which consider the cultural and religious contexts of the target population, are crucial for promoting healthier behaviors and improving public health outcomes across different societies.

Relationship between eHLS and HLSUS

The study revealed a significant positive association between eHLS and HLSUS (β = 0.323), indicating that eHealth literacy influences university students’ health behaviors. Multiple studies have revealed a positive correlation between eHealth literacy levels and health-promoting behaviors among university students. For instance, students with high eHealth literacy tend to actively engage in various health-promoting behaviors, such as better nutritional habits, regular exercise, and effective stress management [37-39]. Previous studies on the impact of eHealth literacy on nursing students’ health-promotion behaviors have shown that low eHealth literacy can lead to difficulties in finding appropriate health information, selecting reliable information, making healthy decisions, and understanding health information. Consequently, this can result in reduced self-management abilities and difficulties maintaining healthy lifestyles [20]. Furthermore, improvements in eHealth literacy have been reported to be associated with a reduced risk of common health issues, such as depression [40], suggesting potential contributions to enhanced mental health. However, barriers such as the complexity of information, inadequate training, and limited access to digital resources have been identified as factors hindering the advancement in eHealth literacy among students [41]. Our findings revealed that improving eHealth literacy could lead to enhanced individual health behaviors. This implies that as eHealth literacy improves, individuals may be more likely to engage in healthy behaviors, such as regular exercise and balanced diets, potentially contributing to NCD prevention.

Previous studies on the relationship between university students’ health behaviors and NCDs have reported sex differences in health behaviors among university students, with male students living alone having a higher NCD risk [42] and female students generally showing higher health consciousness and better health-related habits [43]. As our study focused on nursing students, with >90% of the respondents being female in both countries, the eHLS and HLSUS scores may have been higher than those in other university departments. However, female university students have also been reported to have distorted body image perceptions, engage in inappropriate dieting behaviors, and lack exercise [44]. These findings suggest the need for comprehensive programs that address not only exercise but also dietary knowledge and nutritional balance when developing interventions to improve health behaviors. In addition, a study on smoking, a cause of NCDs, found that smoking rates were overwhelmingly higher among male college students [45] [46] and that self-denial and stress temperament were common factors contributing to smoking [47]. This study may not have obtained accurate data on sex and smoking because of the low proportion of male students and because the survey was conducted only among nursing students. Therefore, considering sex, living arrangements, smoking habits, and health status when intervening in the health behaviors of college students is necessary.

Impact of COVID-19

Although our study was conducted during the COVID-19 pandemic, we acknowledge that assessing the relationship between eHealth literacy and the impact of COVID-19 was not the primary focus of our research. However, the data collected in this context provided a valuable opportunity to explore these associations within the framework of nursing education during a global health crisis. The observed indirect effect of eHealth literacy on HLSUS through the impact of COVID-19 was minimal (0.007), suggesting that the relationships between these variables are complex and warrant further dedicated research. A systematic review investigating the role of eHealth literacy in COVID-19 preventive behaviors emphasized the need to enhance eHealth literacy levels [48].

Highlighting that nursing students typically demonstrate higher baseline levels of eHealth literacy due to their educational background and frequent engagement with health-related information resources is also important [49,50]. This characteristic of our study population should be taken into account when interpreting the findings, as it may limit the generalizability of the results to other populations. Future research might benefit from comparing these relationships across different student populations or educational contexts to provide a broader understanding of the role of eHealth literacy in health crisis management.

Need for educational programs

This study’s results suggest that educational interventions aimed at improving eHealth literacy might contribute to enhancing health behaviors among university students. Many university students possess moderate eHealth literacy and skills, with a particular lack of ability to evaluate the quality of online health resources [51]. This suggests the need for improved training to help students discern reliable information. Future studies should investigate the impact of eHealth literacy on other health risk factors and diseases and assess how eHealth literacy could influence health behaviors across different cultures and countries to develop effective programs.

The multi-group simultaneous analysis supported configural invariance but rejected partial metric invariance. Configural invariance determines whether the factor structure and indicator-factor loading patterns are identical in the baseline model [52]. Although configural invariance was established, further improvement in model fit was not achieved. The establishment of configural invariance implies that data collected from each group can be decomposed into the same structure [53]. This suggests that the measurement models used in both countries have the same factor structure with the same indicators corresponding to their respective factors. In other words, a common standard for understanding and evaluating health behaviors appears to have been established between Indonesia and Japan. Similar educational goals and pedagogy can be applied in the development of future educational programs. Therefore, future program designs and goals should be based on shared factors while adapting to cultural and social backgrounds.

Limitations and future directions

Although this study provides valuable insights, several limitations should be considered when interpreting the findings. First, the generalizability of our results might be limited by the demographic characteristics of our study population, which primarily included nursing students. Nursing students typically have higher levels of eHealth literacy and are more engaged with health-related information, which might have not reflected the broader population. Therefore, these findings may not be directly applicable to non-student populations or individuals in different educational contexts. Future studies could benefit from including more diverse populations to better understand how eHealth literacy functions across different demographic groups.

Second, the study relied on self-reported data, which might have been influenced by social desirability bias, particularly in Indonesia, where religious norms could affect the disclosure of certain lifestyle behaviors. This might result in participants underreporting behaviors perceived as socially undesirable, thus skewing the data. This is a common limitation in studies that rely on self-reported information. Future research might consider using objective measures or alternative data collection methods to mitigate this bias.

Third, the cross-cultural comparability of our findings is an important consideration. The relationship between eHealth literacy and health behaviors might vary across different cultural contexts. Moreover, our study’s results may not be directly applicable to other countries or populations. Therefore, further research should assess these relationships in diverse cultural settings to assess the robustness and applicability of the findings in various global contexts.

Finally, future research should consider reinforcing participant engagement strategies to ensure more consistent and reliable participation, which could help enhance the quality and depth of the data collected.

## Conclusions

eHealth literacy affects the health behaviors of college students in Japan and Indonesia. However, eHealth literacy did not fully mitigate the impact of COVID-19. These results suggest that future educational interventions to promote eHealth literacy may improve the health behaviors of college students.
